# Theoretical Design of Tellurium-Based Two-Dimensional Perovskite Photovoltaic Materials

**DOI:** 10.3390/molecules29133155

**Published:** 2024-07-02

**Authors:** Chunhong Long, Peihao Huang

**Affiliations:** 1 School of Science, Chongqing University of Posts and Telecommunications, Chongqing 400065, China; longch@cqupt.edu.cn; 2 Chongqing Institute of Green and Intelligent Technology, Chongqing School, University of Chinese Academy of Sciences (UCAS Chongqing), Chinese Academy of Sciences, Chongqing 400714, China; 3 University of Chinese Academy of Sciences, Beijing 100049, China

**Keywords:** two-dimensional perovskite, photovoltaic, crystal structure prediction, tellurium-based

## Abstract

In recent years, the photoelectric conversion efficiency of three–dimensional (3D) perovskites has seen significant improvements. However, the commercial application of 3D perovskites is hindered by stability issues and the toxicity of lead. Two–dimensional (2D) perovskites exhibit good stability but suffer from low efficiency. Designing efficient and stable lead–free 2D perovskite materials remains a crucial unsolved scientific challenge. This study, through structural prediction combined with first–principles calculations, successfully predicts a 2D perovskite, CsTeI_5_. Theoretical calculations indicate that this compound possesses excellent stability and a theoretical efficiency of up to 29.3%, showing promise for successful application in thin–film solar cells. This research provides a new perspective for the design of efficient and stable lead-free 2D perovskites.

## 1. Introduction

In 2009, Professor Miyasaka at Toin University of Yokohama in Japan was the first to utilize organic–inorganic hybrid perovskite materials as the light-absorbing layer in solar cells, achieving an efficiency of 3.8% [1]. Since then, 14 years later, perovskite solar cells have attained a certified efficiency of 26.1% [2], matching the efficiency of monocrystalline silicon cells. Compared to conventional crystalline silicon batteries, perovskite batteries have simpler fabrication processes, lower equipment and production costs, as well as an exceptionally lower energy consumption per unit, which can effectively reduce the cost per kilowatt-hour of electricity and carbon emissions. However, the stability issue and the toxicity of lead in these batteries pose hindrances to their commercial application [3].

Recent studies have pointed out that reducing dimensionality is beneficial for the stability of perovskite materials [4,5]. Three–dimensional perovskites are formed by the connection of corner–sharing metal halide octahedrons. By adjusting the organic or inorganic groups in the structure, it is possible to achieve 3D, 2D, 1D, and 0D configurations of perovskites. Their differences in crystal structure, photovoltaic property, and stability are shown in Figure S1 and Table S1. Among these, 2D perovskites are considered the most promising route to overcome the instability of perovskites [6,7,8]. Nevertheless, two-dimensional perovskites exhibit larger optical bandgaps and lower carrier mobility, leading to generally lower device efficiencies. The current highest record for 2D perovskites was achieved with an efficiency of 16.61% in (1–NA)_2_(Cs)_3_Pb_4_I_13_ [9], where the short–circuit current (*J*_SC_) is 19.84 mA/cm^2^. As for 0D perovskite–based cells, the efficiency records are even lower, with the current peak being 3.8%, achieved by using the 0D perovskite (N–EtPy)[SbBr_6_] [10] and *J*_SC_ is 5.1 mA/cm^2^.

Recent theoretical studies have suggested that the 0D perovskite material ATeI_5_ (where A is C_4_H_8_TeI^+^) exhibits both good stability and a high theoretical photoelectric conversion efficiency of 28.7% [11]. However, the photovoltaic performance of the structurally similar 0D perovskite Cs_2_TeI_6_ has been found to be less satisfactory. Cs_2_TeI_6_ exhibits an indirect bandgap of 1.5 eV, absorption coefficients of ~6 × 10^4^ cm^−1^, carrier lifetimes of ~2.6 ns, a work function of 4.95 eV, and *p*–type surface conductivity [12]. Additionally, Cs_2_TeI_6_ is intolerant to the formation of defects, because the defect level occurs deep within the band gap and thus localizes potential mobile charge carriers [13]. As a result, Cs_2_TeI_6_ has not been extensively explored in photovoltaics [14]. This raises questions about the structural characteristics and optoelectronic properties of CsTeI_5_. Through a survey of existing literature, it was discovered that CsTeI_5_ has not yet been reported experimentally. Based on this, our study employed crystal structure prediction methodologies in conjunction with first–principles calculations, successfully predicting a stable crystal structure for CsTeI_5_. Optical property calculations further revealed that its theoretical photoelectric conversion efficiency reaches 29.3%. This research thereby provides a foundation for the design of two-dimensional tellurium–based perovskite photovoltaic materials.

## 2. Results and Discussion

**Figure 1:**
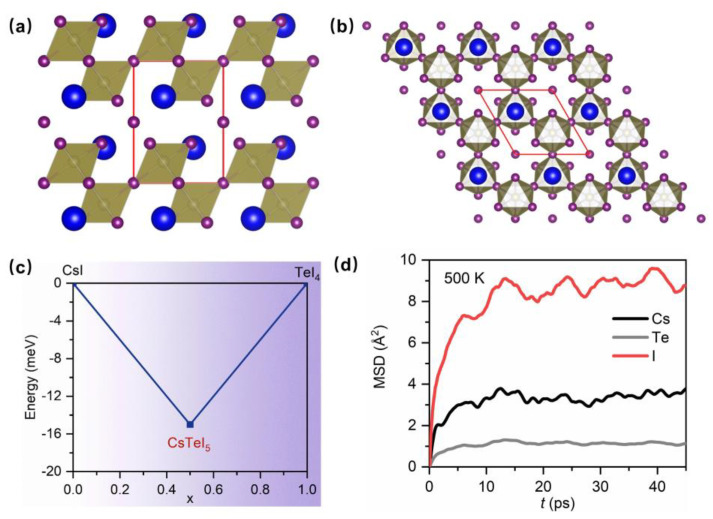
The crystal structure and stability of CsTeI_5_. (**a**) The crystal structure view along the a–axis of CsTeI_5_. The red frame is the primitive cell. (**b**) The crystal structure view along the c–axis of CsTeI_5_. (**c**) The formation energy of CsTeI_5_ relative to CsI and TeI_4_. (**d**) Mean square displacements (MSDs) of CsTeI_5_ at 500 K.

We employed crystal structure prediction methods to investigate CsTeI_5_ within the scope of 1 to 4 times its molecular formula, successfully predicting a stable compound of CsTeI_5_. The compound adopted a *P*-3*m*1 space group, with its structure depicted in Figure 1a,b. The lattice constants of a, b, and c are 9.04 Å, 9.04 Å, and 10.66 Å. In this structure, Te atoms formed an octahedron with I atoms, with each Te–I octahedron connecting to three others, constructing a two-dimensional layered structure of a Te–I octahedron. Cs atoms resided in the intra-layer voids, while the remaining Cs atoms occupied the inter-layer gaps. To study the thermodynamic stability of CsTeI_5_, the typical decomposition path, CsTeI_5_ → CsI + TeI_4_, was considered. The formation energy of synthesizing CsTeI_5_ from CsI and TeI_4_ was calculated, as illustrated in Figure 1c, yielding a value of −15 meV/atom. This negative formation energy indicated the thermal stability of CsTeI_5_. To further examine its dynamic stability, phonon spectrum and first–principles molecular dynamics simulations were conducted to explore the compound’s behavior under finite temperature conditions. The phonon spectrum of CsTeI_5_ showed no imaginary frequency at 300 K, indicating that CsTeI_5_ is dynamically stable (as shown in Figure S2). Ab initio molecular dynamics were conducted at 300 K, 400 K, and 500 K. Based on equilibrium trajectory calculations, the mean square displacements for each of the three constituent elements were determined, as shown in Figure 1d and Figure S3. The findings revealed that at 500 K, no diffusion of the elements occurred, illustrating good dynamic stability.

Ideal photovoltaic materials need a suitable bandgap, high and balanced carrier mobilities, small exciton binding energies, and substantial light absorption coefficients, along with favorable defect characteristics and a lengthy carrier diffusion length. In this study, we focused on the first four optical properties.

**Figure 2:**
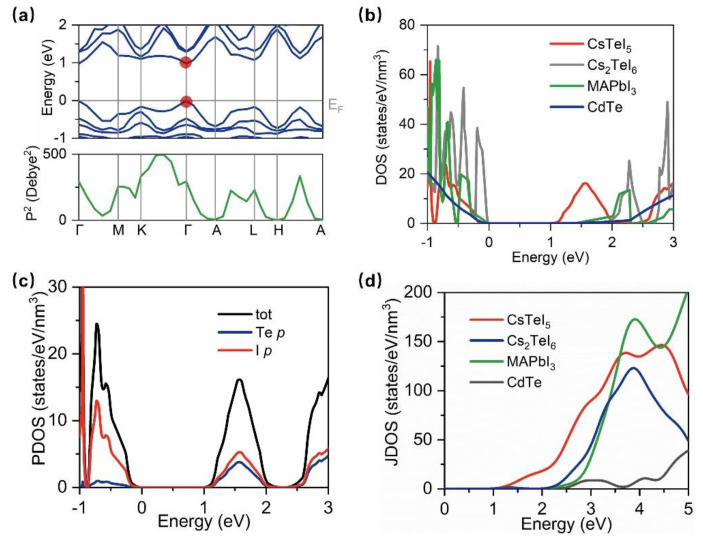
The electronic properties of CsTeI_5_. (**a**) Band structure and transition dipole moment of CsTeI_5_. The VBM and CBM are highlighted as red dots. (**b**) Electronic density of states of CsTeI_5_. (**c**) Projected density of states for CsTeI_5_. (**d**) Joint density of states for CsTeI_5_.

To investigate whether CsTeI_5_ possesses good optoelectronic properties for photovoltaic applications, this study examined the band structure of CsTeI_5_, as depicted in Figure 2a. The band structure was calculated by using the HSE06 functional with the inclusion of SOC, and revealed a direct bandgap with the conduction band minimum (CBM) and valence band maximum (VBM) located at the Gamma point. Direct bandgap materials facilitate photon absorption without phonon assistance, making them ideal for PV applications; typical MAPbI_3_ and CdTe were direct band gap materials, as listed in Table 1. In contrast, Cs_2_TeI_6_ had an indirect bandgap. The calculated bandgap for the *Pnma* phase of MAPbI_3_ in this work is 1.35 eV (HSE + SOC), which is in line with a previous theoretical value of 1.46 eV [15] and an experimental value of 1.5–1.6 eV [16]. The bandgap value of CsTeI_5_ was further computed, yielding a PBE-calculated bandgap of 0.89 eV, which increased to 0.97 eV when employing the hybrid functional, approaching the optimal bandgap of 1.34 eV, as predicted by the Shockley–Queisser limit [17]. As for Cs_2_TeI_6_, it had a much wider bandgap of 1.83 eV and mismatched the visible light spectrum. The electronic density of states (DOS) for CsTeI_5_ showed a high DOS near both the VBM and CBM, which was good for optical absorption (see Figure 2b). To elucidate the orbital contributions at the VBM and CBM, the projected electron density of states (PDOS) was calculated for CsTeI_5_ (see Figure 2c), indicating that the VBM was primarily derived from iodine (I) *p*-orbitals, while the CBM arose from the hybridization of I and tellurium (Te) *p*–orbitals. This aligned with previous findings in systems with lone pair *s*–electrons, where direct bandgap transitions occurred via *p*–*p* orbitals [11]. According to Fermi’s Golden Rule, the absorption coefficient was intimately related to the product of transition matrix elements and the joint density of states (JDOS). The squared transition dipole moment (P^2^) and JDOS were calculated for CsTeI_5_ (Figure 2a,d), demonstrating symmetry–allowed transitions at the Gamma point with high dipole moments. As shown in Figure 2d, CsTeI_5_ exhibited a higher JDOS in the visible light range (1.6–3.2 eV) compared to other materials, suggesting a superior light absorption coefficient. Hence, these results pointed toward CsTeI_5′_s potential for high light harvesting efficiency due to its favorable band structure characteristics and optical properties.

In addition, we investigated the carrier mobility of CsTeI_5_. The relationship between carrier mobility μ, the average free time of carriers τ, and the effective mass $m^{*}$ is expressed as $\;\mu =q\tau /m^{*}$. Due to the computational expense associated with determining τ, we mainly focused on the effective mass $m^{*}$. As presented in Table 1, the effective masses for the hole and electron of MAPbI_3_ are 0.29 *m*_0_ and 0.22 *m*_0_, which is in line with the previous theoretical values of 0.29 *m*_0_ and 0.23 *m*_0_ [18]. The effective masses for the hole and electron of CdTe are 0.58 *m*_0_ and 0.05 *m*_0_, which is in line with previous theoretical values of 0.28 *m*_0_ and 0.09 *m*_0_ [15]. CsTeI_5_ exhibited small and nearly equivalent effective masses for both electrons and holes, comparable to those of MAPbI_3_. From the mobility and effective values, it can be seen that CsTeI_5_ may have large and balanced electron and hole mobility. In contrast, Cs_2_TeI_6_ had a significantly large hole effective mass (0.33 *m*_0_) compared to its electron effective mass (18.39 *m*_0_), which hampered efficient hole transportation.

Furthermore, the static dielectric constant of CsTeI_5_ was calculated and compared with those of Cs_2_TeI_6_, MAPbI_3_, and CdTe. The dielectric constant of MAPbI_3_ in this work is 88.05, which is in line with the previous experimental value of ~70 [19]. The calculated exciton binding energy is 0.2 meV, lower than the experimental value of 2 meV [19]. It was observed that CsTeI_5_, akin to hybrid perovskites, possessed a high dielectric constant of ~200 by using the PBE functional. Since exciton binding energy was inversely proportional to the square of the dielectric constant, this implied that CsTeI_5_ had a minimal exciton binding energy, enabling the thermal energy at room temperature (k_B_T ≈ 27 meV) to readily separate photo-generated excitons into free holes and electrons with minimal energy loss.

**Figure 3:**
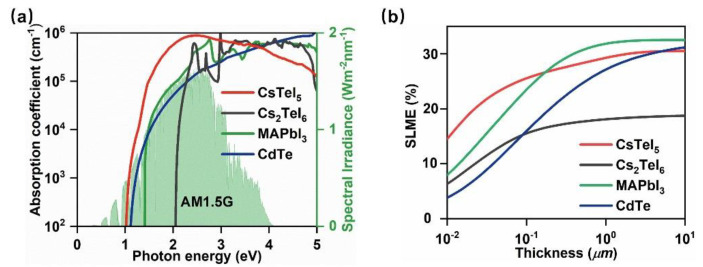
Optical properties. (**a**) Absorption coefficients. (**b**) Theoretical photoelectric conversion efficiency.

Finally, this study calculated the absorption coefficient of CsTeI_5_, as illustrated in Figure 3a. It was evident that across the entire solar spectrum (indicated by the light-green shaded area in Figure 3a), CsTeI_5_ displayed a remarkably high absorption coefficient, exceeding 10^5^ cm^−1^, surpassing even those of perovskites. The advantage of having a high absorption coefficient lies in the ability to absorb the same amount of photons using a thinner film, which can significantly reduce material costs. Utilizing the calculated bandgap, absorption coefficient, and transition dipole moments, the SLME theoretical photovoltaic conversion efficiency limit for a CsTeI_5_–based cell under AM1.5G illumination was simulated, as shown in Figure 3b. The transition dipole moment of CsTeI_5_ at Γ point is 293.25 Debye^2^, indicating that the transition from VBM to CBM is allowed by symmetry. The input data for SLME calculation of CsTeI_5_ are the bandgap (0.97 eV) and absorption coefficient, as shown in Figure 3a, which are calculated at the HSE06 level, with SOC taken into account. Notably, at thicknesses below 100 nm, CsTeI_5_ achieves the highest theoretical efficiency of 25.6%. For thicknesses of 0.5 μm and 1 μm, the theoretical efficiencies slightly dip below those of perovskites, reaching 28.3% and 29.3%, respectively, but still notably higher than CdTe and significantly greater than Cs_2_TeI_6_. Collectively, these findings suggest that CsTeI_5_ is a prospective high–efficiency photovoltaic material, demonstrating exceptional potential for solar cell applications with its combination of high optical absorption, suitable bandgap, and balanced carrier transport properties. Its performance, especially at reduced film thicknesses, underscores its promise for enhancing solar cell efficiency while potentially reducing material usage and manufacturing costs.

## 3. Methods

Below, we illustrated how we predicted the crystal structure and calculated the electronic properties along with the first-principles dynamics simulation setups. In addition, we explained how we calculated the photovoltaic properties, including the exciton binding energy, transition dipole moments, the joint density of states, and the theoretical photoelectric conversion efficiency.

### 3.1. Crystal Structure Prediction and Electronic Properties Calculation

In this work, we adopted the CALYPSO crystal structure prediction method [20,21] and its associated software to predict the crystal structure of CsTeI_5_ under ambient pressure. The CALYPSO approach employs a particle swarm optimization algorithm in conjunction with first-principles calculations for global structure searching. The first–principles calculations were carried out using Vienna Ab initio Simulation Package (VASP 5.4.4) software [22,23], based on the plane–wave pseudopotential method. We utilized the Perdew–Burke–Ernzerhof (PBE) exchange-correlation functional [24] and Projector Augmented Wave (PAW) pseudopotentials [25] for structure optimization and dynamical calculations. A plane–wave cutoff energy of 300 eV and a reciprocal space grid density of 2π × 0.048 Å^−1^ were selected for Brillouin zone sampling. For the first–principles molecular dynamics, an NVT ensemble was employed with a Nosé–Hoover thermostat. The simulation system comprised 112 atoms, with a time step of 2 fs and a total simulation time of 20 ps. The Γ-point was chosen for Brillouin zone k–point sampling during the dynamics simulation. The static dielectric constants were computed using density functional perturbation theory (DFPT). Effective masses of electrons at the conduction band minimum and holes at the valence band maximum were calculated using the finite difference method at these extreme points, which were implemented in EMC (Version 1.0) software.

### 3.2. Photovoltaic Property Calculations

The exciton binding energy was calculated using the formula: $E_{b}=m^{*}R_{y}∕m_{0}\varepsilon^{2}$, where $R_{y}$ is the Rydberg constant and $m^{*}$ is the reduced effective mass calculated as $1/m^{*}=1/m_{e}^{*}+1/m_{h}^{*}$, with $m_{e}^{*}$ and $m_{h}^{*}$ representing the effective masses of electrons and holes, respectively. Given that the PBE functional tends to underestimate band gaps, the HSE06 hybrid functional was further employed to compute more accurate band gap values and absorption coefficients, with spin–orbit coupling (SOC) taken into account. Transition dipole moments and joint density of states were obtained using the VASPKIT GPLv3 [26] software package. To evaluate the photovoltaic performance of the candidate materials, the theoretical photoelectric conversion efficiency was calculated using the Spectral Limited Maximum Efficiency (SLME) method [15].

## 4. Conclusions

Through the application of first–principles–based crystal structure prediction methodologies, this study successfully identified a stable compound of CsTeI_5_, a novel two–dimensional perovskite material. Comprehensive electronic and optical property calculations have illuminated that CsTeI_5_ features a suitable direct bandgap, low effective masses for carriers, minimal exciton binding energy, and high absorption coefficients, thereby marking it as a promising candidate for next-generation two-dimensional perovskite photovoltaics. Remarkably, theoretical calculations estimate its solar–to–electricity conversion efficiency at 25.6% for a thin film thickness of 100 nm, which climbs to 29.3% at a thickness of 1 µm, underscoring its potential for highly efficient energy conversion even at reduced dimensions. Notably, this work did not address the defect properties of CsTeI_5_, which could influence its practical performance, and the actual optical characteristics await experimental validation. Nonetheless, the findings lay a foundation for the development of stable and efficient lead-free perovskite cells, presenting a potential avenue for future integration into printable thin-film solar cells, thereby advancing renewable energy technologies and sustainability efforts.

## Figures and Tables

**Table 1:** Comparison of optical properties.

Compounds	Bandgap (eV)		Effective Mass (*m*_0_)		Dielectric Constant	Exciton Binding Energy (meV)	Direct/Indirect Band Gap	SLME (0.5 μm/L μm)
	PBE	HSE + SOC	$m_{h}^{*}$	$m_{e}^{*}$				
CsTeI_5_	0.89	0.97	0.16	0.18	200.69	0.03	Direct	28.3/29.3
Cs_2_TeI_6_	1.43	1.83	18.39	0.33	10.82	37	Indirect	17.7/18.1
MAPbI_3_	1.77	1.35	0.29	0.22	88.05	0.2	Direct	30.7/31.8
CdTe	0.50	1.05	0.58	0.05	15.39	26	Direct	24.6/27.1

## Data Availability

Dataset available on request from the authors.

## Supplementary Materials

The following supporting information can be downloaded at: https://www.mdpi.com/article/10.3390/molecules29133155/s1.
