# Supplementary material for: Theoretical Design of Tellurium-Based Two-Dimensional Perovskite Photovoltaic Materials
Source: Molecules. 2024 Jul 2;29(13):3155. doi: 10.3390/molecules29133155 (PMC11243364; doi:10.3390/molecules29133155)
Supplement: Supplementary file 1 [file molecules-29-03155-s001.zip › molecules-3073591-supplementary.pdf]

Supporting Information

Theoretical Design of Tellurium-Based Two–Dimensional Perovskite Photovoltaic Materials

Chunhong Long [1] and Peihao Huang [2,3,*]

[1] School of Science, Chongqing University of Posts and Telecommunications, Chongqing 400065, China; longch@cqupt.edu.cn
[2] Chongqing Institute of Green and Intelligent Technology, Chongqing School, University of Chinese Academy of Sciences (UCAS Chongqing), Chinese Academy of Sciences, Chongqing 400714, China
[3] University of Chinese Academy of Sciences, Beijing 100049, China
* Correspondence: huangpeihao@cigit.ac.cn

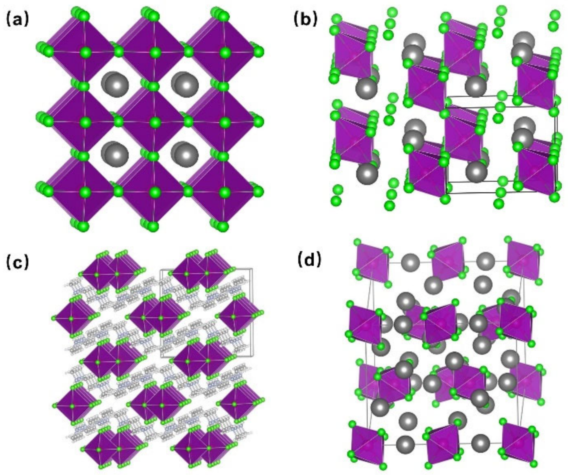

Figure S1. Crystal structures of (**a**) cubic 3D perovskite, (**b**) 2D perovskite, (**c**) 1D perovskite, and (**d**) 0D perovskite at molecular levels.

Table S1. Property comparisons of 3D, 2D, 1D, and 0D perovskites.

**Parameter**	**3D**	**2D**	**1D**	**0D**
Efficiency (%)	26.1%	16.61	-	3.8
V_{OC} (V)	1.174	1.093	-	1.285
J_{SC} (mA/cm^2)	26.13	19.84	-	5.1
FF (%)	85.2	76.63	-	58
E_b (meV)	20-60	100-400	100-500	150-600
Stability	Low	Medium	Medium	High

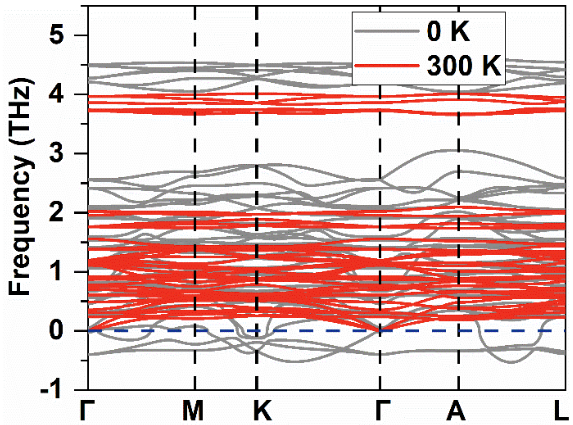

Figure S2. Phonon spectrum of $CsTeI_5$ at 0 K and 300K.

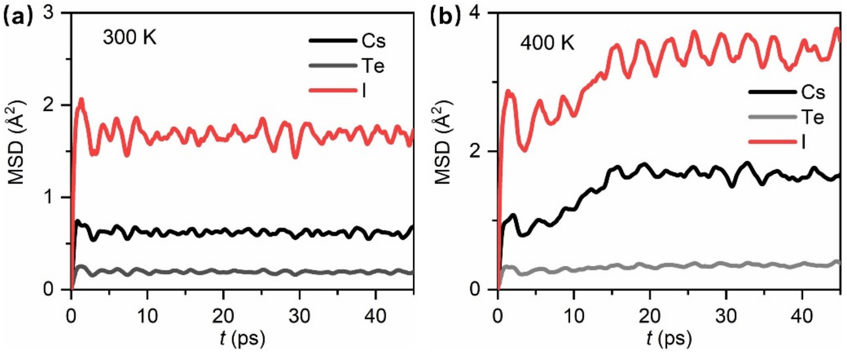

Figure S3. Mean square displacements of $CsTeI_5$ at 300 K and 400 K.

CIF information of CsTeI5

data_CsTeI5 P-3m1
_audit_creation_date 2024-06-24
_symmetry_space_group_name_H-M 'P-3M1'
_symmetry_Int_Tables_number 164
_symmetry_cell_setting trigonal
loop_
_symmetry_equiv_pos_as_xyz
x,y,z
-y,x-y,z
-x+y,-x,z
y,x,-z
x-y,-y,-z
-x,-x+y,-z
-x,-y,-z
y,-x+y,-z
x-y,x,-z
-y,-x,z
-x+y,y,z
x,x-y,z
_cell_length_a 9.0432
_cell_length_b 9.0432
_cell_length_c 10.6621
_cell_angle_alpha 90.0000
_cell_angle_beta 90.0000
_cell_angle_gamma 120.0000
loop_
_atom_site_label
_atom_site_type_symbol
_atom_site_fract_x
_atom_site_fract_y

_atom_site_fract_z
_atom_site_U_iso_or_equiv
_atom_site_adp_type
_atom_site_occupancy

Cs1	Cs	0.66667	0.33333	0.70904	0.01267	Uiso	1.00
Te1	Te	0.66667	0.33333	0.17562	0.01267	Uiso	1.00
I1	I	0.00000	0.00000	0.50000	0.01267	Uiso	1.00
I2	I	0.50000	0.50000	0.00000	0.01267	Uiso	1.00
I5	I	0.36129	0.18065	0.32515	0.01267	Uiso	1.00
